# Incidence of Immune-Related Adverse Events with Program Death Receptor-1- and Program Death Receptor-1 Ligand-Directed Therapies in Genitourinary Cancers

**DOI:** 10.3389/fonc.2017.00056

**Published:** 2017-04-03

**Authors:** Benjamin L. Maughan, Erin Bailey, David M. Gill, Neeraj Agarwal

**Affiliations:** ^1^Huntsman Cancer Institute, University of Utah, Salt Lake City, UT, USA; ^2^Department of Internal Medicine, University of Utah, Salt Lake City, UT, USA

**Keywords:** immunotherapy, checkpoint blockade, toxicity, treatment, autoimmune, immune-related adverse events

## Abstract

Program death receptor-1 (PD-1) and program death receptor-1 ligand (PD-L1) inhibitors are increasingly being used in the clinic to treat a growing number of malignancies, including many genitourinary (GU) malignancies. These immune-based therapies have demonstrated a distinct toxicity profile compared to traditional chemotherapy and the targeted therapies directed at the vascular endothelial growth factor pathway or the mammalian target of rapamycin pathway. Autoimmune toxicity targeting the skin, gastrointestinal tract, or the endocrine organs are some of the more common adverse events (AEs) noted with these therapies. Here in, we report the results of a systematic review of the incidence of toxicities in GU cancers reported in the phase II or phase III clinical trials using single-agent PD-1 or PD-L1 inhibitors. Overall, the rate of serious (grades 3–4) AEs was noted in approximately 15% of patients. The AEs noted were similar between all the agents tested, highlighting the overall class effect of these therapies. The incidence in GU cancers is similar to those seen in other malignancies. Given the widespread and high volume real-world use of these agents, it is important for oncologists to be familiar with these side effects to minimize the risks for patients while undergoing therapy.

## Introduction

The approval of ipilimumab for metastatic melanoma in 2011 spearheaded the development of numerous anticancer therapies targeting immune checkpoint pathways. To date, these novel therapies have been approved in quick succession by the US Food and Drug Administration (FDA) for the treatment of a variety of solid tumors and hematologic malignancies. These therapies have been shown to produce prolonged, durable complete remissions in a subset of patients even with high volume metastatic disease, which was not previously seen with traditional chemotherapy or the targeted therapies. The success of ipilimumab, a monoclonal antibody targeting cytotoxic T-lymphocyte-associated protein 4, led to an explosion of research resulting in the development of new immunotherapy agents, focusing on a variety of new immune targets. The most prominent of these new discoveries are monoclonal antibodies targeting programmed cell death protein 1 program death receptor-1 (PD-1), for example, nivolumab and pembrolizumab, or its ligand program death receptor-1 ligand (PD-L1), for example, durvalumab and avelumab. Many of these PD checkpoint inhibitors are now FDA approved, leading to a significant increase in their clinical use over the last few years. Through the clinical trials already conducted, it is evident that the side effects are quite distinct from traditional chemotherapy and the targeted therapies previously approved. Here, we report the results of a systematic review on the incidence of autoimmune adverse events (AEs) reported in the phase II or phase III clinical trials in genitourinary (GU) cancers using single-agent PD-1 or PD-L1 inhibitors.

## Mechanism of Action of PD-1 and PD-L1 Inhibitors

**Figure 1:**
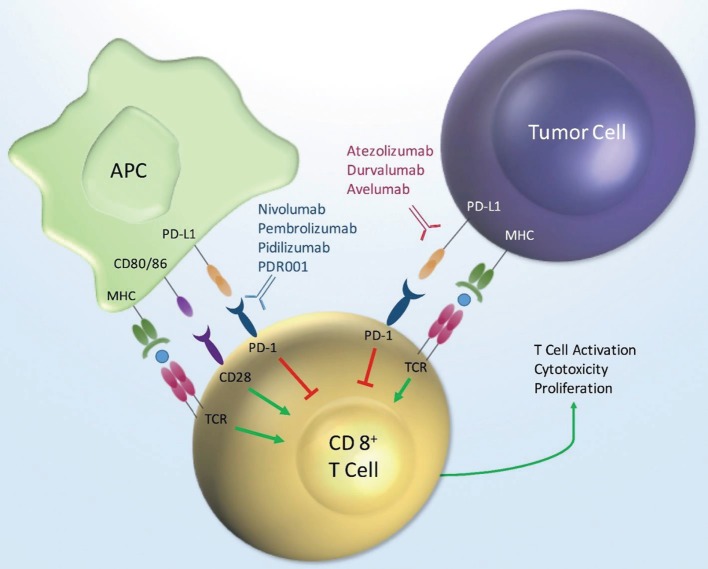
**PD-1/PD-L1 mechanism of action**. Key: MHC, major histocompatibility; APC, antigen presenting cell; PD-L1, programmed death ligand-1; PD-1, programmed death receptor-1; TCR, T-cell receptor.

Program death receptor-1 and PD-L1 are cell surface proteins that are part of the large immunoglobulin superfamily. Antagonists of these receptors are included in the diverse drug class referred to as immune checkpoint inhibitors. The PD-1 and PD-L1 antagonistic antibodies are designed ultimately to augment the immune system as a mechanism of treating cancer. Treatment directed against these targets directly leads to activation of cytotoxic T-lymphocytes (Figure 1). The mechanism of action for checkpoint inhibitors and the clinical efficacy of these therapies in GU and other malignancies have been reviewed previously in detail (1–5). Briefly, activation or suppression of T cells requires a two-step process. First, the antigen presenting cell (APC), such as dendritic cells, processes and presents the antigen to T-cells *via* the major histocompatibility complex (MHC) *via* direct binding to the T-cell receptor (TCR). In addition to the interaction of MHC and TCR, a second co-stimulatory or co-suppressor signal is required for an appropriate immune response. PD-1 is a receptor found on many immune mediator cells such as T-cells, natural killer cells, dendritic cells, and B-cells (6). PD-L1 is one of two known ligands (PD-L1 and PD-L2) for PD-1, and both are co-inhibitory to T-cell activation. PD-L1 is found on APCs as well as tumor cells (7). The co-inhibitory signal from PD-1 and PD-L1 signaling provides the necessary inhibitory signal, driving the T-cell into a state of inactivity.

## Clinical Activity of PD-1 or PD-L1 Checkpoint Inhibitors in GU Cancers

These agents have demonstrated significant activity in GU malignancies including renal cell carcinoma (8, 9) and urothelial carcinoma, leading to approval of some of these agents (10). Additionally, trials investigating many of these agents in the advanced prostate cancer are ongoing.

In the seminal report on the efficacy of nivolumab (8), patients with metastatic renal cell carcinoma were randomly assigned in 1:1 fashion to treatment with nivolumab or everolimus after prior progression on treatment with vascular endothelial growth factor receptor tyrosine kinase inhibitors. Eight hundred twenty-one patients were enrolled. The median overall survival was 25.0 months (95% CI, 21.8–not estimable) for nivolumab compared to 19.6 months (95% CI, 17.6–23.1 months) for everolimus, with a hazard ratio of 0.73 (98.5% CI, 057–0.93, *P* = 0.002) favoring nivolumab therapy. The objective response rate was 25 versus 5% (OR 5.98; 95% CI, 3.68–9.72; *P* < 0.001) for nivolumab and everolimus, respectively. These results have recently led to approval by the FDA of nivolumab in this setting.

In May 2016, atezolizumab was FDA approved for urothelial carcinoma based on the results of a phase II clinical trial (10). Patients with inoperable locally advanced or metastatic platinum-refractory urothelial carcinoma were treated with atezolizumab 1200 mg every 3 weeks until disease progression or dose-limiting toxicity. Three-hundred ten patients were treated in this single-arm study and stratified by the percent positivity of PD-L1 staining on the tumor infiltrating lymphocytes (Group 1 is <1%; Groups 2 is >1% but <5%; and Group 3 is ≥5%). The objective response rates were 15, 18, and 26%, respectively, for groups 1–3. All groups had improved rates of objective responses compared to historic controls (10%), including a 6–11% complete response rate.

Based on these encouraging results, and the subsequent FDA approvals, multiple other PD-1 or PD-L1 checkpoint inhibitors are in clinical trials as single-agent therapies or in combination with other antineoplastic therapies including pembrolizumab (PD-1 inhibitor), durvalumab (PD-L1 inhibitor), avelumab (PD-L1 inhibitor), atezolizumab (PD-L1 inhibitor), and PDR001 (PD-1 inhibitor). Here, the authors review the immune-related side effect profiles of PD-1 and PD-L1 inhibitors in GU malignancies.

## Inconsistencies in the Definition of Immune-Related Adverse Events (irAEs) Across PD-1 or PD-L1 Inhibitor Trials

Currently, there is no guideline or consensus on how to define and report irAEs in clinical trials. This has resulted in lack of consistency among various clinical trials in reporting the incidence, onset, and duration of AEs. This does create problems when comparing AEs across the trials. For instance, diarrhea and colitis are reported separately in the studies discussed in this review, and the definition for colitis varies between these studies. In the studies with atezolizumab (10–12), irAEs are defined as those events requiring systemic corticosteroids and with no other identifiable underlying cause. By contrast, in one nivolumab study (13), irAEs were defined as any toxicity with a potential immune-mediated etiology, which may or may not have required special monitoring and specific unique interventions. In another nivolumab study (14), reports of irAE were restricted to events requiring use of an immune-modulating therapy, with the exception of endocrine events. In the prescribing information for nivolumab (15) immune-related pneumonitis, nephritis, colitis, and hepatitis were required by definition to have no alternate etiology and necessitated treatment with systemic corticosteroids. The durvalumab trial did not have a formal requirement for systemic corticosteroids, but categorized events as immune-related if the AE was consistent with an immune-mediated mechanism of action and there was no clear alternate etiology (16). The authors were unable to identify the definition for irAE in the study with pembrolizumab (17). The differences in definitions for irAE among trials involving PD-1 and PD-L1 therapies create hurdles in accurately interpreting the data. There are also other dissimilarities between the reporting of toxicities with irAE and non-immune AEs between these studies. Standardization of the definition of irAE will likely improve the interpretation of clinical trials for clinicians, patients, and policy makers in the future.

## Methods

A systematic review of the literature was performed according to the Preferred Reporting Items for Systematic Reviews and Meta-Analyses recommendations (18). In October 2016, PubMed, International Pharmaceutical Abstracts (IPA), and EMBASE databases were queried for relevant published literature on all phase II or phase III studies with any PD-1 or PD-L1 therapy in GU malignancies (prostate, urothelial, testicular, and renal cell carcinoma). The search terms may be found in Presentation S1 in Supplementary Material. All database queries were limited to human trials published in the English language. Additionally, the prescribing information for FDA-approved PD-1 or PD-L1 therapies was reviewed. Finally, conference abstracts, posters, and platform presentations from 2016 ESMO and ASCO Meetings were queried for updated published material. Published literature was excluded if the trial involved non-genitourinary malignancies, as well as all phase I studies. Trials involving combination anticancer therapy were excluded; however, concomitant hormonal therapy was allowed. Manufacturers of FDA approved therapies for GU malignancies (nivolumab and atezolizumab) were consulted for Supplementary Material on the outcome of interest when published materials were incomplete.

## Results

### Search Results

**Figure 2:**
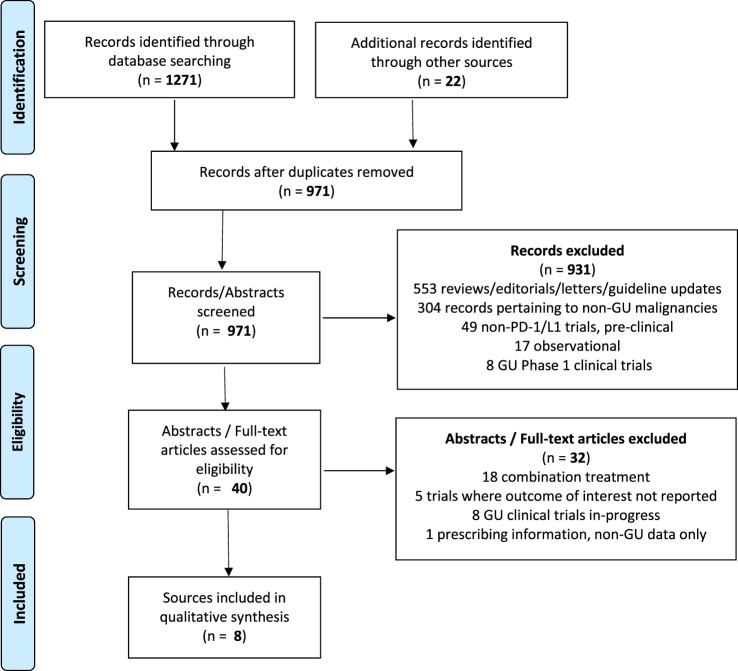
**Data selection process (18)**.

A total of 931 citations were identified through EMBASE, 283 citations in PubMed, and 57 abstracts through the IPA. In total, 969 unique studies were identified. After removing the citations for combination trials, published data where outcomes of interest were not reported, trials involving non-genitourinary malignancies and non-primary literature (reviews, guideline updates, etc.), there were eight sources that met criteria for study inclusion (Figure 2).

### Atezolizumab

One clinical trial was identified, with a total of 429 patients with urothelial carcinoma, which evaluated atezolizumab treatment in a single-arm, two cohort study (10–12). Atezolizumab was given as a 1200 mg intravenous infusion every 3 weeks until dose-limiting toxicities or until loss of clinical benefit to all patients. Cohort 1 (*n* = 119) included patients without prior treatment for metastatic disease and were ineligible for cisplatin-based chemotherapy. Cohort 2 (*n* = 310) included patients with metastatic disease who progressed in platinum therapy. The irAE incidence was 12% (14 patients) for all grades, 7% (8 patients) for grades 3–5 in cohort 1 (11). This was similar to cohort 2, with 10% (31 patients) for all grades and 6% (19 patients) for grades 3–5 (12). The most common irAEs were rash, transaminitis, rhabdomyolysis, pneumonitis, and hyperbilirubinemia. The most frequent grades 3–5 irAEs were transaminitis and hyperbilirubinemia. Pneumonitis, endocrinopathies, and rhabdomyolysis were noted with ≤1% incidence for grades 3–5 toxicities (Table 1).

**Table 1:** **Incidence of immune-related adverse events (irAEs) by treatment type for genitourinary (GU) cancers in phase II/III Trials (11–14, 16, 17, 19, 22)**.

Program death receptor-1 (PD-1)/program death receptor-1 ligand (PD-L1) inhibitor	Trial	Dose	*n*	GU cancer	All grades irAEs	G3–5 irAEs
Atezolizumab (PD-L1)	IMVigor 210 (cohort 1), phase II	1200 mg	119	Urothelial	**Total 14 (12%)** Rash, *n* = 4 (3%) Increased ALT, *n* = 2 (2%) Increased bilirubin, *n* = 2 (2%) Rhabdomyolysis, *n* = 2 (2%) ≤1%: Increased AST, liver disorder, colitis, autoimmune colitis, diarrhea, hypothyroidism, arthralgia, maculopapular rash, arthritis, rheumatoid arthritis, muscle spasms, tenosynovitis	**Total 8 (7%)** Increased ALT, *n* = 2 (2%) Increased bilirubin, *n* = 2 (2%) ≤1%: Increased AST, liver disorder, rash, rhabdomyolysis, colitis, autoimmune colitis, diarrhea, rheumatoid arthritis
	
	IMVigor 210 (cohort 2), phase II^b^	1200 mg	310	Urothelial	**Total 31 (10%)** Pneumonitis, *n* = 6 (2%) Increased AST, *n* = 6 (2%) ≤1%: Increased ALT, transaminases increased, dyspnea, colitis, diarrhea, rash, increased bilirubin, dry skin, pruritus, pyrexia, hyperglycemia	**Total 19 (6%)** ≤1%: Pneumonitis, increased AST, increased ALT, transaminases increased, increased bilirubin, dyspnea, colitis, diarrhea, rash, autoimmune hepatitis, hepatitis, cytokine release syndrome, paraplegia, pericardial effusion, alkaline phosphatase increased, chronic kidney disease, hypotension, musculoskeletal pain, sepsis

Durvalumab (PD-L1)	Durvalumab, phase I/II	10 mg/kg	61	Urothelial	**Total 14 (23%)** Diarrhea, *n* = 6 (9.8%) Pruritus, *n* = 2 (3%) Infusion-related reactions, *n* = 2 (3.3%) Nephritis (biopsy proven), *n* = 1 (1.6%) *Other*: *n* = 4 total other irAEs occurred in 1 patient each (1.6% each)	**Total 2 (3%)** Nephritis (biopsy proven), *n* = 1 (1.6%) Infusion-related reactions, *n* = 1 (1.6%)

Nivolumab (PD-1)	CheckMate 025, phase III^a^	3 mg/kg	406	Renal	**Total: NR** Hypothyroidism, *n* = 33 (8.1%) Rash, *n* = 30 (7.4%) Infusion-related reactions, *n* = 25 (6.2%) Pneumonitis, *n* = 18 (4.4%) Diarrhea/colitis, *n* = 13 (3.2%) Renal dysfunction/nephritis, *n* = 12 (3%) Hyperthyroidism, *n* = 10 (2.5%) Adrenal insufficiency, *n* = 8 (2%) Diabetes, *n* = 6 (1.5%) Hepatitis, *n* = 6 (1.5%) ≤1%: Hypophysitis	**Total: NR** Renal dysfunction/nephritis, *n* = 5 (1.2%) Pneumonitis, *n* = 5 (1.2%) Diarrhea/colitis, *n* = 5 (1.2%) Hepatitis, *n* = 5 (1.2%) ≤1%: Rash, adrenal insufficiency, diabetes, hypothyroidism, hypophysitis
	
	Nivolumab, phase II	0.3, 2, and 10 mg/kg	167	Renal	**Total: NR** Skin, *n* = 40 (24%) Endocrine, *n* = 18 (11%) GI, *n* = 17 (10%) Hypersensitivity, *n* = 11 (7%) Pulmonary, *n* = 9 (5%) Hepatic, *n* = 9 (5%) ≤1%: Renal	**Total: NR** Hepatic, *n* = 3 (2%) ≤1%: Skin, endocrine, GI
	CheckMate 032, phase I/II	3 mg/kg	78	Urothelial	**Total: NR** Rash, *n* = 7 (9%) Hypothyroidism/thyroiditis, *n* = 7 (9%) Hyperthyroidism, *n* = 3 (4%) Hepatitis, *n* = 3 (4%) Pneumonitis, *n* = 2 (3%) Diarrhea/colitis, *n* = 2 (3%) ≤1%: Nephritis/renal impairment, hypersensitivity	**Total: NR** Hepatitis, *n* = 3 (4%) ≤1%: Pneumonitis (worsened to G5), diarrhea/colitis, nephritis/renal impairment, rash

Pembrolizumab (PD-1)	Pembrolizumab, phase II	200 mg	20	Prostate	**Total 5 (25%)** Colitis, *n* = 2 (10%) Hypothyroidism, *n* = 2 (10%) Myositis, *n* = 1 (5%)	**Total 3 (15%)** Colitis, *n* = 2 (10%) Hypothyroidism, *n* = 1 (5%)

Pembrolizumab (PD-1)	KEYNOTE-052, phase II	200 mg	100	Urothelial	**Total: NR** Hypothyroidism, *n* = 6 (6%) Pneumonitis, *n* = 3 (3%) ≤1%: Nephritis, colitis	**Total: NR** Pneumonitis, *n* = 2 (2%) ≤1%: Nephritis, colitis

*^a^CheckMate 025: permission obtained to use data from nivolumab website: http://www.opdivoyervoyhcp.com/servlet/servlet.FileDownload?file=00Pi000000SRPWfEAP*.

*^b^IMvigor 210 (cohort 2): reported all grades irAEs occurring in ≥2 patients, reported grades 3–4 irAEs occurring in ≥1 patient*.

### Durvalumab

One clinical trial was identified, which included 61 patients with urothelial carcinoma who declined, or were ineligible for, or had progressed after platinum-based chemotherapy (16). Durvalumab was given as a 10 mg/kg intravenous infusion every 2 weeks for up to 12 months. The irAE incidence was 23% (14 patients) experiencing any grade of toxicity, and 3% (2 patients) with a grade 3–5 toxicity. The most common irAEs were diarrhea, pruritus, and infusion-related reactions. The two grade 3–5 reactions were biopsy-proven nephritis and an infusion-related reaction (Table 1).

### Nivolumab

Three clinical trials were identified. In the first study, nivolumab was compared to everolimus in a randomized trial in patients with metastatic renal cell carcinoma who had progressed on one or two antiangiogenic therapies (8). Nivolumab was given as an intravenous infusion at 3 mg/kg every 2 weeks with a total of 406 patients treated in the nivolumab arm. The overall incidence of all grades of AE rate was 79%, and 19% of patients experiencing grade 3–4 events. The overall incidence of irAE was not reported. The second study involving 168 metastatic renal cell carcinoma patients tested three different doses of nivolumab in patients who progressed on antiangiogenic therapy. Nivolumab was administered as an intravenous infusion at 0.3, 2, or 10 mg/kg every 3 weeks (13). In the safety population (*n* = 167), the overall incidence of any grade AE in the 0.3 mg/kg treatment arms was 75%, and 5% for grades 3–4. For the 2 mg/kg arm, the incidence for any grade was 67%, with 17% experiencing grades 3–4 reactions. For the 10 mg/kg arm, the incidence for all grades was 78%, with 13% experiencing a grade 3–4 reaction. Again, the overall incidence of all irAE was not reported. In the third study, nivolumab was given intravenously every 2 weeks at 3 mg/kg in 78 patients with urothelial carcinoma who previously progressed on platinum-based chemotherapy (14, 23). The total number of irAE was not reported. The incidence for any AD for all grades was 81% with 22% of patients experiencing grade 3 events. Notably, 1 patient experienced a grade 5 event (pneumonitis, patient died). Pneumonitis, colitis, transaminitis, hypothyroidism, and rash were the most commonly reported grades 3–4 irAEs. Hypophysitis, adrenal insufficiency, diabetes mellitus, hyperthyroidism, and nephritis were also reported (Table 1).

### Pembrolizumab

Pembrolizumab, a PD-1 antagonist, was given as a 200-mg flat dose intravenous infusion every 3 weeks for four doses in patients with metastatic castration-resistant prostate cancer, previously progressing on enzalutamide (17). Patients could not have received prior chemotherapy. There were 20 patients treated on this study. Any grade of irAE occurred in 25% of patients (5 patients) and grade 3 irAE occurred in 15% of patients (3 patients). The most common irAEs of any grade were myositis (*n* = 1), hypothyroidism (*n* = 2), and colitis (*n* = 2). The three grade 3 events were hypothyroidism (*n* = 1) and colitis (*n* = 2). There were no grade 4 or 5 events reported (Table 1).

The KEYNOTE-052 study (19) in cisplatin-ineligible patients evaluated pembrolizumab in the first-line setting in urothelial carcinoma in 200 patients. Pembrolizumab was given as 200 mg IV every 3 weeks. The treatment related AE for any grade was 67 and 15% for grades 3–4 events. The overall incidence for irAE was not reported. Pneumonitis was noted in 3% of patients, nephritis in 1%, colitis in 1%, and hypothyroidism in 6%.

The irAEs reported in all of these studies with GU cancers, including the incidence of grades 3–5 toxicities, are similar to those reported in trials of PD-1 inhibitors in non-genitourinary clinical trials. Thus, the incidence of irAE in GU cancers appears to be representative of the overall cancer patient population (Table 2).

**Table 2:** **Comparison of selected immune-related adverse event (irAE) in GU versus non-genitourinary clinical trials (11–17, 19–22)**.

Adverse event	Incidence, any grade (GU only trials) (%)	Incidence, grades 3–5 (GU only trials) (%)	Incidence any grade (non-GU clinical trials) (%)	Incidence, grades 3–5 (non-GU clinical trials) (%)
Hypothyroid/thyroiditis	0.8–9	0–0.6	3.9–12	0–0.1
Diabetes/DKA	0–1.5	0–0.7	0.8–0.8	0.4–0.7
LFT changes/hepatitis	1.5–5.4	1–3.8	0.3–3.4	0.3–2.7
Pneumonitis	2–4.4	0–2	1.8–3.5	0.25–1.9
Encephalitis	NR	NR	0.2–0.8	0.0–0.2
Colitis/diarrhea	1–10	1–10	2.4–4.1	1.0–2.5
Hypophysitis^a^	0–0.5	0–0.2	0.2–0.9	0.2–0.4
Renal Dysfunction/nephritis	0.3–1.6	0–1.6	0.3–4.9	0.0–0.5
Myositis^a^	0.8–5	0–0.8	NR	NR

*GU, genitourinary; DKA, diabetic ketoacidosis; LFT, liver function test; NR, not reported*.

*^a^Reported in only one study*.

## Underreporting of AEs

Recent analysis of clinical trials involving immune checkpoint therapy, including PD-1 and PD-L1 therapies, highlights the potential concern of underreporting of irAEs. Chen et al (24) evaluated all clinical trials involving any checkpoint inhibitor and noted large variability in the quality of irAE reporting. The authors used a 21-point oncology specific AE reporting score based on the CONSORT criteria as the quality benchmark. The median incidence of grades 3–4 toxicities was 22%, however, with a range of 0–66%. The review identified overall poor reporting of toxicity management (8%), reversibility of AE (6%), and the onset of the AE (14%). These findings are similar to an updated analysis that was reported this year. Bossi et al. (25) analyzed immunotherapy and targeted therapy clinical trials for the quality of AE reporting, also benchmarked against the CONSORT criteria. More than 90% of clinical trials scored poorly in their reporting of recurrent and late toxicities, and in the duration of the AEs. This is a consistent finding between these two analyses. Most trials did not report on the time to the AE occurrence (86%), and only reported on AEs that occurred at a frequency above a fixed threshold (75%). Dose reductions due to AE were not reported in one-third of trials.

If studies are only reporting irAE at a specific threshold, or not fully reporting on the extent of the disease complications, then the potential risk with this therapeutic class might be significantly underappreciated. This issue is particularly relevant given the diversity in reported AEs and the generally low incidence of any one specific irAE from PD-1/PD-L1 therapies. In aggregate though, the overall incidence of any significant (grades 3–4) irAE is relatively frequent (16–22%) as reported in the clinical trials examined in this review. This is consistent with the reported grades 3–4 AE rate in clinical trials across all cancers (24).

All the reported evidence to date of PD-1 and PD-L1 inhibitors in GU malignancies are based on clinical trials. There are no reports of AEs from a real-world experience. This distinction is important as the complication rates may be higher in general practice compared to the clinical trial experience (26–28). However, some studies document comparable incidences of AEs between clinical trials and the real-world experience (29, 30). It is possible that the incidence of irAE and AE will be higher for PD-1 and PD-L1 inhibitors than is reported in these clinical trials. This increased incidence could be related to less experience with the safe use of these agents between study investigators and community practice providers given the unique type of irAEs, the strict inclusion/exclusion criteria of clinical trials and the rare or delayed nature of the irAEs.

Familiarity with the complications of treatment from immunotherapy is important for all oncologists. The indications for these therapies are continuing to expand, and use of these treatments is expected to continue to grow rapidly over the next few years. Currently, there are three FDA-approved PD-1 or PD-L1 agents [nivolumab, pembrolizumab, and atezolizumab (15, 20, 21)], two of which have indications for use in GU malignancies (nivolumab and atezolizumab). In contrast, there are over 500 clinical trials in all cancers (31). Likely, some of these newer agents will obtain FDA approval, and the number of clinical indications will expand. Treating oncologists will likely be using these therapies more frequently and thereby, encounter the irAEs frequently. This is a rapidly evolving area in GU oncology with many new clinical trials opening. New results are constantly being published in the literature and presented at professional meetings each year. With many novel therapies and new immunotherapy combinations being studied, it will remain important to be aware of the incidence and prevalence of irAEs in GU malignancies.

## Treatment of irAE

Treatment of any irAE caused by PD-directed therapies is largely determined by the severity as defined in the Common Terminology Criteria for Adverse Events (CTCAE) criteria. This validated tool is not specifically designed for immunotherapy, leading some people to question the utility of this tool (24). The CTCAE does not adequately capture the duration of therapy or the irreversibility of an AE, both of which are important considerations in treatment planning. The CTCAE may be modified in the future to better reflect the specific complications seen with immunotherapy. Still, this is a reasonable tool in identifying the severity of illness and therefore guiding the clinician in taking the appropriate steps for initiating treatment.

With all AEs encountered during immunotherapy treatment, it is important to rule out other causes in all cases. Frequently, multidisciplinary treatment is required for both a thorough workup and appropriate treatment, especially regarding serious (grades 3–4) irAEs. Oftentimes, this approach is most effectively performed at referral centers with subspecialty care. The majority of serious AEs are reversible with discontinuation of immune therapy and appropriate treatment. For that reason, corticosteroid therapy generally can be safely tapered over approximately 1 month after the symptoms begin to improve. Endocrinopathies are a notable exception, as these AEs can be permanent, potentially requiring life-long therapy (32, 33).

Treatment algorithms developed previously for the management of ipilimumab therapy have been reported (34, 35). There are no trials or reported experiences evaluating treatment approaches specifically for PD-1- or PD-L1-mediated irAEs. The treatment algorithm for all checkpoint blockade therapy is based on the experience with ipilimumab (22).

## Limitations

In addition to the issue of inconsistent reporting of the irAEs in these trials, as described above, we were unable to find published data that met the inclusion criteria of phase II or phase III studies for avelumab (PD-L1 antagonist), PDR001 (PD-1 antagonist), and pidilizumab (presumed PD-1 antagonist). In addition, the sample size was small for durvalumab. Because the reported irAEs are similar between all the PD-1 and PD-L1 antagonists reported to date, the AEs and irAEs discussed here appear to be a drug class effects and not simply related to a single compound. Therefore, we expect that other PD-1 and PD-L1 directed therapies, such as pidilizumab, are likely to have a similar incidence of irAEs. Given the consistency of the toxicity profile with GU malignancies compared to other malignancies, the immune-mediated events do not appear to be significantly related to disease type.

The Keynote-045 study was recently reported at the SITC meeting in November 2016 (after the date cutoff in this systematic review). This study provides additional safety information related to pembrolizumab. This trial includes an additional 270 patients treated with pembrolizumab. When published, these data will provide significantly more safety data for this agent in urothelial cancers.

## Conclusion

Overall, the incidence of serious (grades 3–4) AEs was noted in approximately 3–15% of GU cancer patients treated in these phase II and III monotherapy trials of PD-1 or PD-L1 checkpoint inhibitors. The reporting for AE and irAE was different between the studies, limiting the accuracy of identifying the true incidence for irAE. Although, the most common organ sites of toxicity include the skin, endocrine organs, lung, and the gastrointestinal tract, any organ can be affected. The AEs noted were similar between all the agents tested, highlighting the overall class effect of these therapies. The irAEs seen in patients with GU cancers were similar to those seen in patients with other cancers. It is well established that incidence of irAEs with checkpoint inhibitors in the clinical trials is underreported. Additionally, the real-world use of these drugs is likely going to be widespread, and the real-world patients likely will be frailer with more comorbidities than the clinical trial patients. This suggests that the overall magnitude of the irAEs of PD-1 and PD-L1 inhibitors are expected to be much higher in the real-world setting. Increased awareness, and timely recognition and management of these toxicities are expected to reduce risk and improve clinical benefit with this class of agents.

## Author Contributions

BM and EB contributed equally to the preparation of this manuscript and compiled the database search for this project. BM, EB, DG, and NA wrote the manuscript and made revisions to the manuscript, tables, and figures. BM, EB, and DG created tables and figures.

## Conflict of Interest Statement

The authors declare that the research was conducted in the absence of any commercial or financial relationships that could be construed as a potential conflict of interest.
