# Supplementary material for: Incidence of Immune-Related Adverse Events with Program Death Receptor-1- and Program Death Receptor-1 Ligand-Directed Therapies in Genitourinary Cancers
Source: Front Oncol. 2017 Apr 3;7:56. doi: 10.3389/fonc.2017.00056 (PMC5377000; doi:10.3389/fonc.2017.00056)
Supplement: Supplementary file 1 [file Presentation_1.PDF]

**Supplementary Appendix: Search Terms**

Embase search string was:

- 'atezolizumab'/exp OR 'avelumab'/exp OR 'durvalumab'/exp OR 'nivolumab'/exp OR pdr001 OR 'pembrolizumab'/exp OR 'pidilizumab'/exp AND [humans]/lim AND [english]/lim AND [embase]/lim AND
    - 'bladder cancer'/exp
        - Returns: 174 abstracts/full texts screened
    - 'germ cell tumor'/exp
        - Returns: 9 abstracts/full texts screened
    - 'kidney carcinoma'/exp
        - Returns: 462 abstracts/full texts screened
    - 'prostate cancer'/exp
        - Returns: 286 abstracts/full texts screened

PubMed search string was:

- (((("pidilizumab"[Supplementary Concept] OR "pembrolizumab"[Supplementary Concept]) OR "nivolumab"[Supplementary Concept]) OR "MPDL3280A"[Supplementary Concept]) OR "avelumab"[Supplementary Concept]) OR "MEDI4736"[Supplementary Concept] OR PDR001 AND ("humans"[MeSH Terms] AND English[lang])

International Pharmaceutical Abstracts (IPA) search string was:

(atezolizumab or avelumab or durvalumab or nivolumab or pdr001 or pembrolizumab or pidilizumab).sh.Limits applied: (English language and human and ("abstracts of meeting
